# Supplementary material for: Breaking the Fear Barrier: Aberrant Activity of Fear Networks as a Prognostic Biomarker in Patients with Panic Disorder Normalized by Pharmacotherapy
Source: Biomedicines. 2023 Aug 29;11(9):2420. doi: 10.3390/biomedicines11092420 (PMC10525800; doi:10.3390/biomedicines11092420)
Supplement: Supplementary file 1 [file biomedicines-11-02420-s001.zip › biomedicines-2535296-supplementary.pdf]

Figure S1: Violin plots depicting demographic and clinical data for all participants.

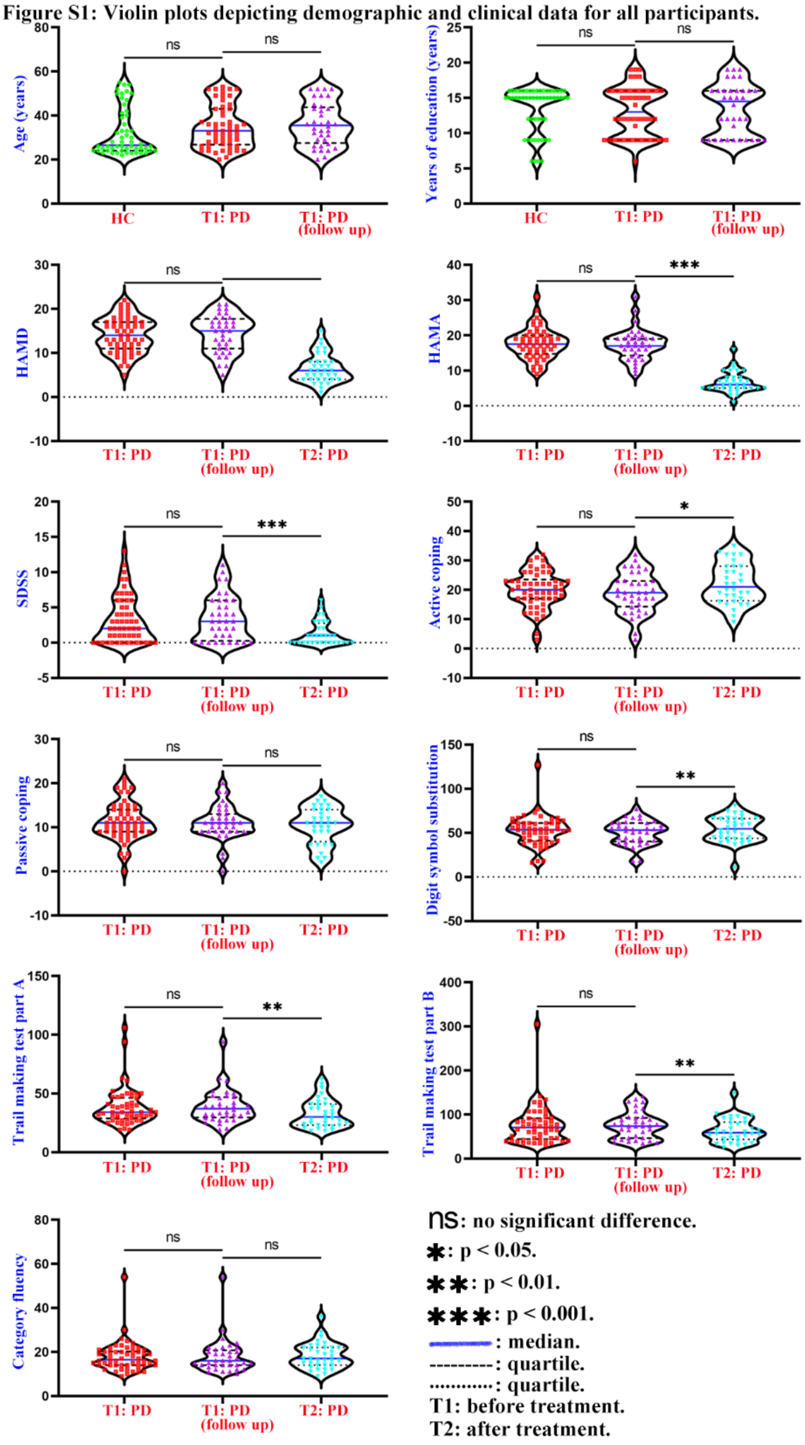

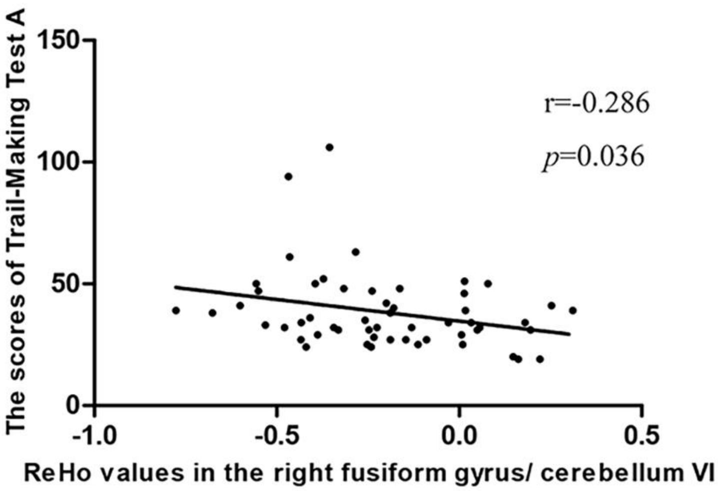

Figure S2: Abnormal ReHo values in right fusiform gyrus/cerebellum VI were negatively correlated with the scores of TMT-A (r = −0.286, p = 0.036).

Table S1. Characteristics of patients who finished the follow-up.

Variables	Pre-treatment (Mean ± SD, n=36)	Post-treatment (Mean ± SD, n=36)	*Z/T*	*p*	*df*	*r*/Cohen's d
Age (years)	36.06±9.75					
Sex (male/female)	16/20					
Years of education (years)	13.42±3.59					
Illness duration (months)	17.83±25.51					
Time of treatment (days)	34.81±7.09					
HAMD	14.32±4.28	6.47±3.29	-5.09	<0.001[a]	35	-0.62
HAMA	17.32±4.49	6.68±2.95	-5.09	<0.001[a]	35	-0.62
SDSS	3.50±2.96	1.31±1.69	-3.90	<0.001[a]	35	-0.47
CSQ						
Active coping	19.34±6.93	22.06±6.93	2.51	0.017[b]	35	0.39
Passive coping	10.81±4.09	10.41±4.13	-0.51	0.611[b]	35	-0.10
B-CATS						
Digit symbol substitution	49.85±13.86	54.24±14.49	3.35	0.002[b]	35	0.31
Trail making test part A	38.94±14.55	33.06±11.95	-2.64	0.008[a]	35	-0.32
Trail making test part B	74.62±28.94	63.79±26.86	-2.90	0.004[a]	35	-0.35
Category fluency	17.94±7.78	18.29±5.91	-1.38	0.167[a]	35	-0.17

[a] The *p*-values were obtained by Wilcoxon signed-rank tests.

[b] The *p*-values were obtained by paired *t*-tests.

SD = standard deviation; HAMD = Hamilton Depression Rating Scale; HAMA = Hamilton Anxiety Rating Scale; SDSS = Social Disability Screening Schedule; CSQ = Simplified Coping Style Questionnaire; B-CATS = Brief Cognitive Assessment Tool for Schizophrenia.
